# Study on heat transfer law of moving temperature variable gas in thermoacoustic plate stack

**DOI:** 10.1038/s41598-024-60293-2

**Published:** 2024-04-25

**Authors:** Jianxin Wang, Xiangbin Liu

**Affiliations:** School of Mechanical Engineering, Inner Mongolia University of Science and Technology, Baotou, 014010 China

**Keywords:** Thermoacoustic refrigeration, Variable temperature gas, Heat transfer process, Mathematical model, Critical frequency, Statistical physics, thermodynamics and nonlinear dynamics, Thermodynamics

## Abstract

Taking gas and the heat transfer process between gas and plate as the research object, the mathematical model of heat transfer in one working cycle by moving variable temperature air mass under the action of sound field is established, which provides a new idea for understanding thermoacoustic effect. The influence factors in the model are analyzed and it is found that the amplitude of the air mass has a significant influence on the heat transfer, and the heat transfer of the air mass in one working cycle is proportional to the square of the amplitude. In a certain working environment, the thermoacoustic refrigerator has a critical operating frequency, and only when the operating frequency is greater than the critical frequency can refrigeration be realized. The critical operating frequency is independent of the amplitude and increases with the increase of the stack temperature gradient. With the pressure belly point as the reference position, the greater the distance from the reference position, the greater the critical operating frequency. On this basis, the idea of short plate overlapping is put forward and the formation mechanism of temperature difference between two ends of plate overlapping is explained.

## Introduction

Due to the destructive effect of traditional refrigeration technology on the environment, people have carried out a lot of research on alternative technologies and new environmentally friendly refrigerants. Among them, thermoacoustic refrigeration system highlights the huge development potential with its advantages of low cost and environmental friendliness. In addition to traditional sound sources (loudspeakers), thermoacoustic refrigerator can also be driven by heat engines powered by waste heat or solar energy^1–3^. The cooling process is realized by the thermodynamic cycle of gas between plates, and there are no moving parts in the resonator, which is reliable and easy to maintain, which is another advantage of thermoacoustic refrigerator. After decades of exploration, the basic theory and practical application have been greatly developed, but there are still problems such as low efficiency of thermoacoustic refrigerator and incomplete basic theory.

The current thermoacoustic theory was proposed by Rott^4–6^, Later, Swift, Wheatley et al.^7,8^ explained the working process and refrigeration principle of the thermoacoustic refrigerator in detail, and established the linear thermoacoustic theory under certain assumptions, which was also the main theoretical basis for the later design of thermoacoustic refrigerator. However, the linear theory has a large number of parameters, and to optimize a single factor or a few easy to operate factors, it is necessary to carry out a large number of calculations, often using the computer program DeltaE to simulate. If in the early stage of the development of thermoacoustic refrigerator, a more simplified calculation method can be used to analyze the correlation between the working parameters and the design objective (the temperature difference between the two ends of the plate or the cooling capacity), it will effectively improve the efficiency of the design stage. Therefore, it is of great significance for the development of thermoacoustic technology to study the heat transfer process between gas and plate, analyze the heat mechanism of micro-air mass pump heat,and find the relationship between plate and plate heat flow and working parameters.

Heat transfer between gas and plate in thermoacoustic refrigerator is a very complicated physical process. In terms of refrigeration principle, relevant literature has explained the heat transfer mechanism from the thermodynamic cycle process of the micro-air mass^9,10^, qualitatively described the temperature change process of the micro-air mass, and studied the effects of various parameters on the refrigeration performance under different working conditions combined with experiments; Worlikar et al.^11–13^ studied the thermal stratified flow near idealized thermoacoustic plate stack through numerical simulation; Cao et al.^14^ established a one-dimensional ideal plate heat transfer model and simulated the thermoacoustic system by solving a complete two-dimensional Navier–Stokes equation; Ishikawa and Mee^15^ further simulated on the basis of Cao 's work, and calculated the energy flux density, particle path and entropy generation rate by using different lengths of plates; The research team of Qing ^16–18^ established a microcycle model of thermoacoustic refrigeration from the perspective of quantum mechanics, and analyzed the refrigeration coefficient and refrigeration rate. Based on this, Anqing Shu^19,20^ optimized the performance of the microcycle model, and the results provided a new research method for thermoacoustic theory; The research team of Syeda Humaira Tasnim^21^ calculated the flow field and thermal field in the thermoacoustic refrigerator, and obtained the change law of the flow and temperature field near the thermoacoustic plate stack.

After 2000, more research was done on the development and performance optimization of thermoacoustic refrigerator under specific working conditions. The relationship between cooling temperature and cooling capacity and working parameters was found by experiment. Hariharan and Sivashanmugam^22^ adopted the curved surface response method and took the temperature difference between the two ends of the stack as the design goal, optimized the stack position, stack length, working frequency and other factors, and obtained a temperature difference of 40K at the working frequency of 254Hz; Nor Atiqah Zolpakar et al.^23^adopted multi-objective genetic algorithm to optimize the performance of thermoacoustic refrigerator, analyzed the effects of stack length, stack center position and plate spacing on the performance of thermoacoustic refrigerator, and conducted experimental verification. Ahmed I. Abd El-Rahman^24^ designed a thermoacoustic refrigerator driven by two pistons. The working medium is air, the displacement amplitude of the driver is 19mm, the working frequency is 42Hz, and the maximum temperature difference between the two ends of the plate stack is 27°. Islam ^25^ studied the influence of piston displacement amplitude and cold side temperature on refrigeration performance in a small thermoacoustic refrigerator, and obtained the result that the cooling capacity and temperature difference increased with the increase of displacement amplitude, but the refrigeration coefficient decreased slightly. Emanuele Sarpero et al.^26^ used 3D printing technology to design different plate stack structures. When the operating frequency was 192 Hz, the maximum temperature difference between the two ends of the plate stack was 18.42 K.

Most of the literature on the pumping heat process of gas on the plate is analyzed through the thermodynamic process of gas microclusters, mainly the qualitative explanation. The details of heat conduction process between microair mass and plate stack and the mechanism of plate stack temperature difference are not elaborated. However, the calculation and measurement show that the temperature change of the air mass in a working cycle is very small, but after the system reaches a stable state, the temperature difference between the two ends of the stack can be quite considerable. Based on this, the details of heat transfer process between microair mass and plate stack are studied in this paper. gas is considered as a mobile variable temperature heat source. The physical and mathematical models of heat transfer between the moving temperature variable gas and the plate under the action of sound field are established. For the first time, the heat transfer between the gas microclusters and the plates during a working process is quantitatively calculated. At the same time, the idea of short plate stack series combination is put forward, ( the whole plate stack can be regarded as a series of several short plate stack),and the mechanism of large temperature difference between the two ends of plate stack is explained. The influence factors in the mathematical model are analyzed, and the variation of air mass moving heat and critical working frequency with working parameters is obtained. The research results provide a new way to understand the thermoacoustic effect and improve the cooling capacity of thermoacoustic refrigerator.

## Calculation of heat transfer by air mass vibration in one cycle

**Figure 1:**
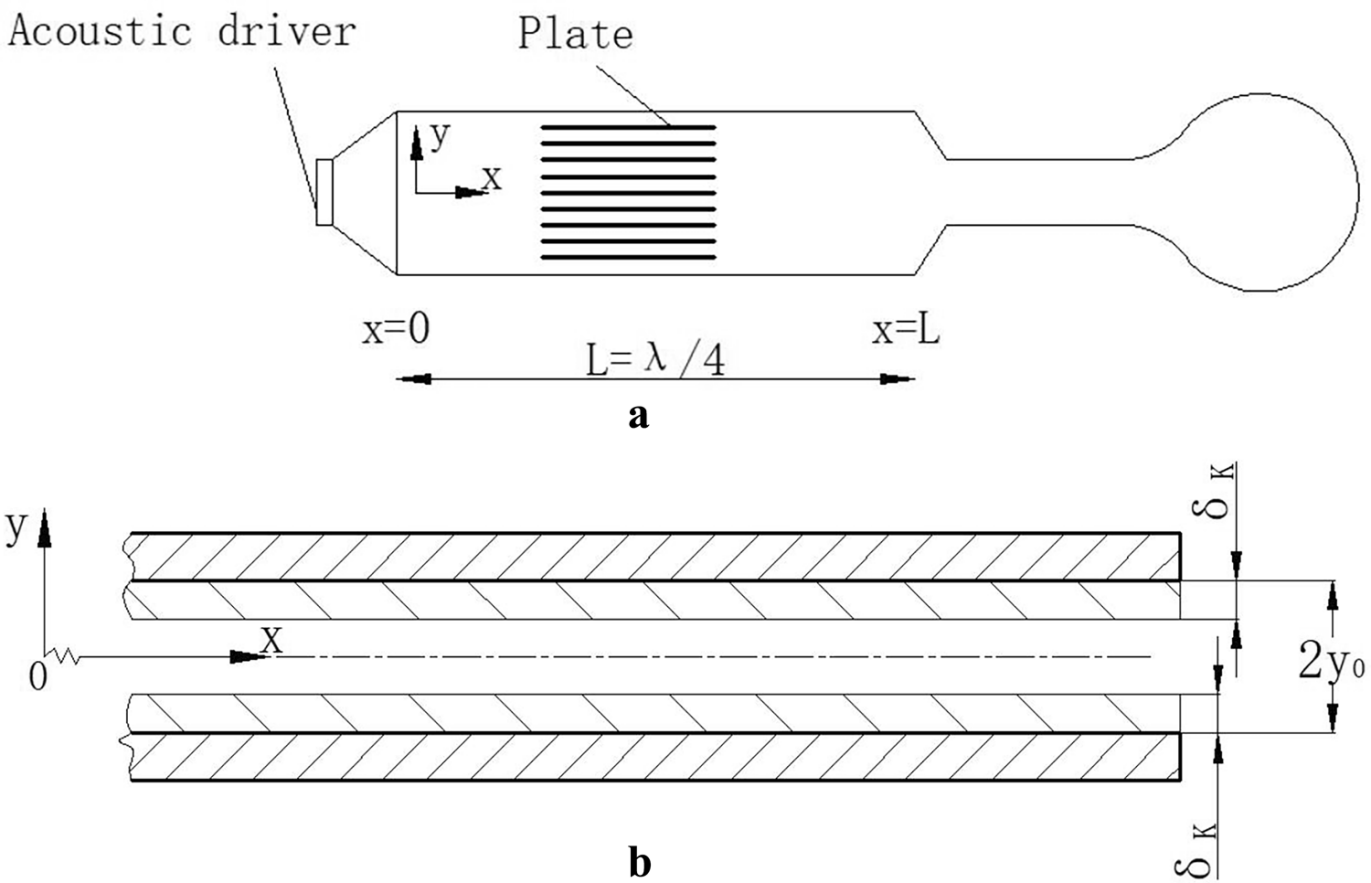
Structure diagram of thermoacoustic refrigerator: (**a**) schematic diagram of structure principle, (**b**) enlarged view of the plate segment.

For the standing wave thermoacoustic refrigerator, the gas between the wave belly and the node has the same motion state, and the gas microcluster on the same section has the same motion direction and phase. The phase difference between pressure and velocity is 90°, both a function of position and time, and pressure is in phase with temperature. Fig 1a shows a schematic diagram of the structure principle of a typical standing wave thermoacoustic refrigerator. In order to study the law of heat transfer by microair mass on the stack, a coordinate system as shown in Fig 1b was established, taking the gas between any two plates as the research object, in which the direction of sound wave propagation is x forward, perpendicular to the surface of the stack is y direction, the coordinates of the upper and lower surfaces of the plate are ± y_0_, and the sound pressure node near the sound source is the coordinate origin.

To quantify the amount of heat moved by an air mass vibrating at its equilibrium position for one week, make the following basic assumptions:

1. It is assumed that the air mass has the same temperature in a plane perpendicular to the direction of movement and is in phase with the pressure;
2. The width of the stack has no influence on the thermoacoustic effect, and the heat conduction problem on the stack can simplify the two-dimensional heat conduction problem;
3. At the beginning of each working cycle, the air mass is in the equilibrium position, and the surface temperature of the sheet is consistent with the temperature of the gas;
4. Do not consider viscosity loss.

**Figure 2:**
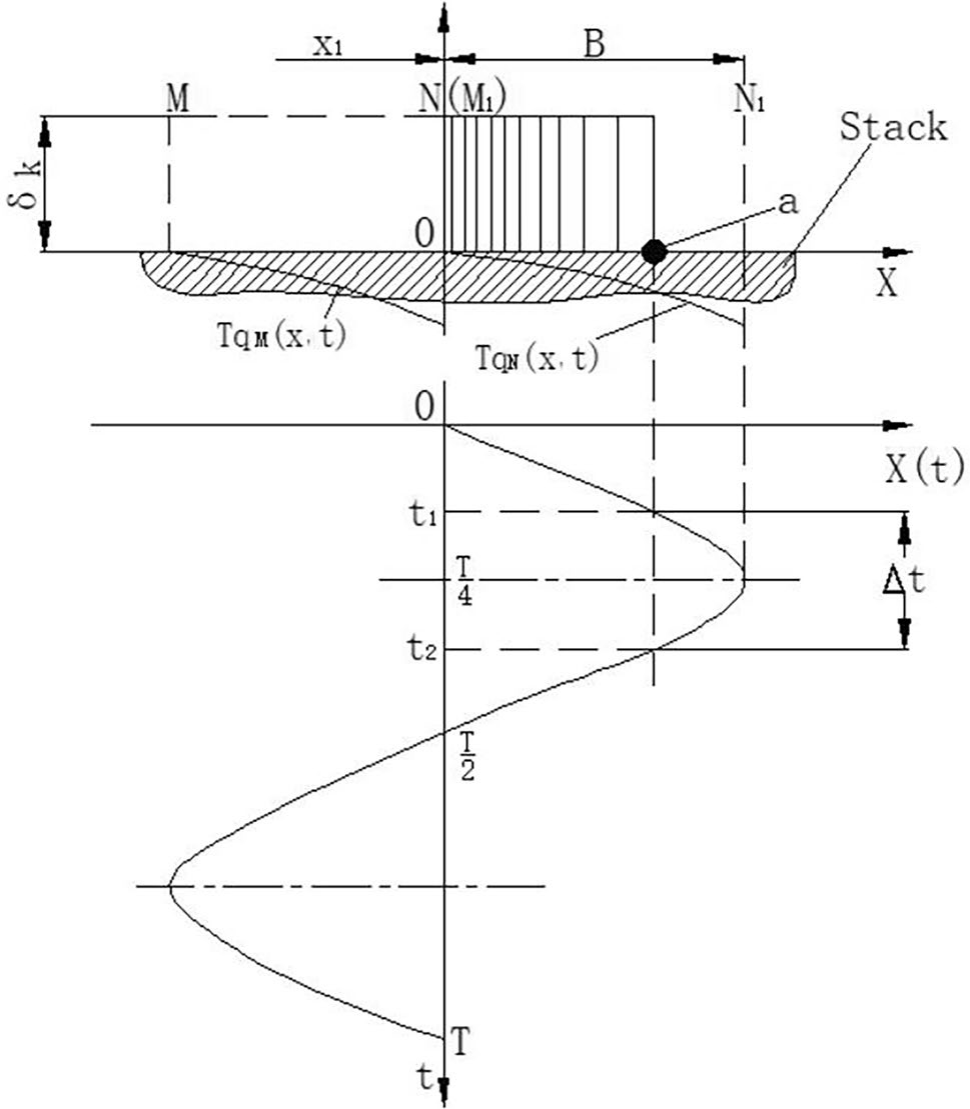
Physical model of heat transfer by microair mass.

The plates in the resonator are parallel plates, and the gas movement between the plates and the energy exchange behavior of the upper and lower plates are the same. The convection heat transfer process between a single plate and the air mass above it is studied. To this end, a small air mass is taken on the surface of the stack, the thickness is the thermal penetration depth, the length is an amplitude, the right side of the air mass is located at N point, the left side is located at M point, and the origin position of the N point distance coordinate is x1. The physical model of heat transfer by microair mass is shown in Fig 2. At the beginning, the right side of the air mass moves to the right from point N, and the whole air mass is like a continuous moving air column extending to the right from the equilibrium position. Since the amplitude of the gas column is small relative to the wave length, deformation during movement is negligible. After moving an amplitude, the right side of the air mass reaches point N_1_, and the left side reaches point M_1_. The farther the air mass is from the equilibrium position, the lower the pressure, the thinner the gas, and the lower the temperature. After moving an amplitude, the right side of the air mass reaches point N_1_, and the left side reaches point M_1_. The farther the air mass is from the equilibrium position, the lower the pressure, the thinner the gas, and the lower the temperature. The temperature at each point can be expressed as a function of displacement and is in phase with the pressure, and the temperature is considered to be uniform in the direction perpendicular to the plate surface in the depth of thermal penetration. The air mass acts as a source of variable temperature heat moving continuously over the surface of the plate, the length is one amplitude.

In Fig. 2, ${T}_{qM}(x,t)$ represents the temperature change curve of the left end face of the air mass moving from point M to point M_1_, and ${T}_{qN}(x,t)$ represents the temperature change curve of the right end face of the air mass moving from point N to point N_1_. In the process of gas movement, the duration of heat transfer between the gas and a point a on the stack is $\Delta t={t}_{2}-{t}_{1}$, within an amplitude range, where ${t}_{1}$ is the time for the right end of the gas column to reach point **a** from the starting position, and ${t}_{2}$ is the time for the right end of the gas column to reach the limit position from the starting position and return to point **a**.

According to literature^9^, the temperature distribution of the gas between the refrigerator plates can be expressed as follows:

1 $${T}_{1}=\bigg(\frac{\gamma -1}{\gamma }\frac{{T}_{m}}{{P}_{m}} {p}_{1}-\frac{1}{j\omega }{u}_{1}\frac{d{T}_{m}}{dx}\bigg)\bigg(1-\frac{\mathit{cos}h\left[\frac{\left(1+j\right)y}{{\delta }_{k}}\right]}{\mathit{cos}h\left[\frac{\left(1+j\right){y}_{0}}{{\delta }_{k}}\right]}\bigg)$$

where: ${T}_{m}$ is the environment temperature, ${\text{K}}$; ${P}_{m}$ is the initial gas pressure, ${\text{pa}}$; ${{\text{p}}}_{1}$ is the sound pressure, ${\text{pa}}$; ${u}_{1}$ is the air mass vibration velocity, ${\text{m}}/{\text{s}}$; $\gamma$ is the specific heat ratio of gas; $\frac{d{T}_{m}}{dx}$ is the temperature gradient of the panel, ${\text{K}}/{\text{m}}$; ${\delta }_{k}=\sqrt{\frac{2K}{\omega \rho {c}_{p}}}$ is the heat penetration; $K$ is the thermal conductivity of gas, ${{\text{w}}\,\cdot ({{\text{m}}}^{2}\cdot\mathrm{ K})}^{-1}$; $\rho$ is the gas density, ${\text{kg}}/{{\text{m}}}^{3}$; ${c}_{p}$ is the specific heat capacity of gas at constant pressure, ${\text{J}}\, \cdot{(\mathrm{kg\cdot K})}^{-1}$; $\omega$ is the angular frequency of gas vibration, ${\text{rad}}/{\text{s}}$.

The second factor to the right of the equal sign in Eq. (1) represents the temperature distribution of the gas in the y direction, which is related to the stack structure. swift explains the stack of different structures in thermoacoustic engines and refrigerators^12^, and gives the corresponding temperature distribution expression. When y = y_0_, the value of T_1_ on the stack surface is zero, and the farther away from the stack surface, the greater the value of T_1_. When the distance is greater than δ_K_, T_1_ gradually becomes stable^12,20^. According to the basic assumption, there is no temperature gradient of the gas in the y direction, and the heat transfer between the gas and the plate is convection. Formula (1) can be simplified as follows:

2 $${T}_{1}=\bigg(\frac{\gamma -1}{\gamma }\frac{{T}_{m}}{{P}_{m}} {p}_{1}-\frac{1}{j\omega }{u}_{1}\frac{d{T}_{m}}{dx}\bigg)$$

The first term on the right of Eq. (2) indicates the gas temperature change caused by sound pressure, and the second term indicates the gas temperature change caused by the stack temperature gradient. According to the thermodynamic theory, the temperature change caused by sound pressure in the process of gas vibration is very small. For example, when the environmental pressure is 1 standard atmospheric pressure, the sound pressure is 145 pa (sound pressure level 137 dB), and the ambient temperature is 300 K, the temperature change of the gas calculated from the first item of equation (2) is about 0.12 °C, so people will not notice the thermoacoustic effect in life.

**Figure 3:**
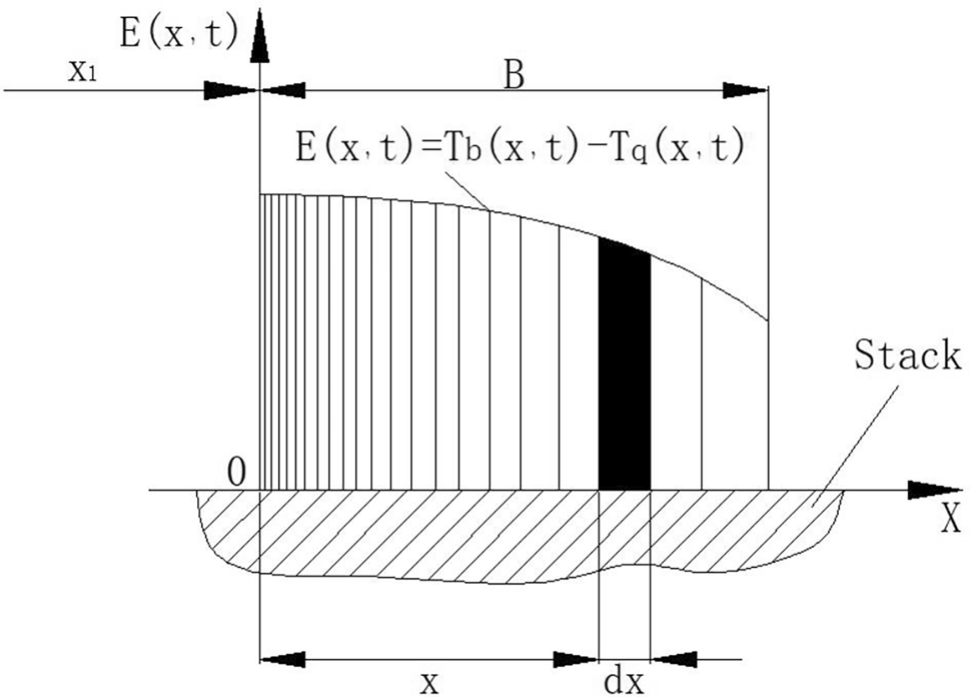
Endothermic calculation model of microair mass on the right side of equilibrium position.

The gas moves on the plate stack. According to the convection heat transfer theory, the heat exchanged between the plate stack and the gas per unit area in a unit time can be expressed as:

3 $$Q=h({T}_{b}-{T}_{q})$$

h is the convective heat transfer coefficient, Tb plate stack temperature, Tq gas temperature. In Eq. (3), Tb and Tq are functions of position and time, and the calculation of heat transfer requires a microelement analysis method. Therefore, the coordinate system as shown in Fig. 3 is established.

Take an arbitrary micro heat source of width dx at a location x away from the origin. The temperature can be considered uniform in the dx range (the trapezoid with curved edge is approximately rectangular). Assuming that the interface temperature difference between the plate stack and the gas is E(x,t) = T_b_ (x,t) − T_q_ (x,t), the micro heat absorbed by the micro-heat source gas (dx) per unit width from the plate stack within the micro-time dt of interaction with the plate stack is:

4 $$dq=hE(x,t)\cdot dx\cdot dt$$

In the process of the right side of the air mass moving from the reference point to the right to the limit position and then returning to the reference point, the heat absorbed from the plate in a region of amplitude is:

5 $$q=h{\int }_{0}^{B}dx{\int }_{{t}_{1}}^{{t}_{2}}\left[{T}_{b}(x,t)-{T}_{q}(x,t)\right]\cdot dt$$

Since $x=Bsin2\pi ft$, can be obtained:

6 $${t}_{1}=\frac{1}{2\pi f}arcsin\left(\frac{x}{B}\right)$$

where *f* is the vibration frequency. The heat transfer time between a and gas on the plate stack is $\Delta {\text{t}}=2(\frac{T}{4}-{t}_{1})$,as shown in Fig. 2. Where T is the period of gas vibration. The motion process of the air mass from the initial position to the right limit position and from the right limit position to the initial position is symmetric about the ${\text{t}}=\frac{T}{4}$ axis. Therefore, to solve the heat absorbed by the air mass from a plate of amplitude B on the right side of the reference point, integrate in the interval t_1_ ~ $\frac{T}{4}$ and then multiply by 2, that is:

7 $$q=2h{\int }_{0}^{B}dx{\int }_{{\text{t}}1}^{\frac{{\text{T}}}{4}}\left[{{\text{T}}}_{{\text{b}}}({\text{x}},{\text{t}})-{{\text{T}}}_{{\text{q}}}({\text{x}},{\text{t}})\right]\cdot {\text{dt}}$$

At the beginning of each working cycle, the gas at each point is located at its own equilibrium position, and it can be considered that the temperature distribution law of the plate and the gas contact surface is the same, and the two have the same initial temperature. When the air mass moves from the initial position to the right limit position, the temperature difference generated by the contact surface is the temperature change of the air mass, namely:

8 $${{\text{T}}}_{1}={T}_{b}(x,t)-{T}_{q}(x,t)$$

Equation (2), (6) and (8) are substituted into Eq. (7) to calculate the heat absorbed from the plate stack during the movement of the air mass on the right side of the reference position:

9 $$q=2h{\int }_{0}^{B}\frac{1}{\omega }\left[\frac{\gamma -1}{\gamma }\frac{{T}_{m}}{{P}_{m}}{ p}_{1}-B\frac{{dT}_{m}}{dx}\right]\sqrt{1-{(\frac{x}{B})}^{2}}dx$$

It can be seen from Eq. (1) that the variation of air mass temperature is the result of the joint action of sound field and plate temperature gradient. The amplitude of air mass is much smaller than the wavelength, so ${p}_{1}$ can be regarded as a constant value in terms of amplitude, and Eq. (9) can be simplified as follows:

10 $$q=2h\frac{1}{\omega }\left[\frac{\gamma -1}{\gamma }\frac{{T}_{m}}{{P}_{m}}{ p}_{1}-B\frac{{dT}_{m}}{dx}\right]{\int }_{0}^{B}\sqrt{1-{(\frac{x}{B})}^{2}}dx$$

The heat transferred by the air mass in one working cycle is the heat absorbed by the air mass moving on the right side of the reference position. By calculating formula (10), it is obtained that the heat transferred by the air mass from the plate stack in one working cycle is:

11 $$q=\frac{\pi }{2}{\delta }_{k}\Pi h\frac{1}{\omega }B\left[\frac{\gamma -1}{\gamma }\frac{{T}_{m}}{{P}_{m}}{ p}_{1}-B\frac{{dT}_{m}}{dx}\right]$$

By substituting ${p}_{1}=\rho c\omega B$ into Eq. (11), we get:

12 $$q=\frac{\pi }{2}{\delta }_{k}\Pi h{B}^{2}\left[\frac{\gamma -1}{\gamma }\frac{{T}_{m}}{{P}_{m}}\rho c-\frac{1}{\omega }\frac{{dT}_{m}}{dx}\right]$$

Formula (12) is the mathematical model of the heat transferred from one side to the other side during a working cycle of the gas microcluster, where $\Pi$ is the width of the plate stack. According to formula (12), the heat transferred is proportional to the cross-sectional area ${\delta }_{k}\Pi$ of the gas involved in heat transfer above the plate stack, and the larger the cross-sectional area, the more gas involved in the work, the more heat moved; It is proportional to the convective heat transfer coefficient; It is proportional to the square of the gas amplitude, which can be understood from two aspects: on the one hand, the increase of the amplitude can increase the sound field intensity and increase the temperature change of the air mass; on the other hand, the increase of the amplitude increases the length of the air mass on the plate and its surface, and expands the heat transfer area.

As the heat transfer process continues, the temperature of the stack and its contact surface with the gas micromass will change until a stable temperature gradient is formed when the heat transferred by the microair mass is balanced with the heat leakage of the stack. A high temperature heat source is formed at the left end of the stack, and a cold source is formed at the right end of the stack.

The heat flux between plates in a thermoacoustic system is the heat transferred from the low temperature end to the high temperature end per unit length of gas in a unit time multiplied by the speed of gas movement. By calculating formula (12), the heat flux between plates of the thermoacoustic refrigerator can be obtained from the heat moved by the microair mass in one working cycle:

13 $${Q}_{c}={n}_{q}{n}_{t}Q\cdot {v}_{q}$$

where: ${Q}_{c}$ is the heat flux between plates; ${n}_{q}$ is the number of microair masses per unit length of gas; ${n}_{t}$ is the number of times the microair mass moves heat per unit time; ${v}_{q}$ is the speed of the air mass.

The development process of thermoacoustic refrigerator is reported in detail in reference^20^, and the working parameters set are shown in Table 1. When the cold end temperature is 229 K and the frequency is 409 Hz, the heat flow rate in the resonator is 2.75 W. Bring the parameters in reference^20^ into Eqs. (12) and (13), it can be obtained that the heat moved by the micro-air mass in one working cycle and the heat flux between the panels of the thermoacoustic refrigerator are 0.0018 W and 2.594 W, respectively. The calculated results are slightly different from those in reference^20^, and are not obvious. Nsofor et al.^28^found in the experimental study on the performance of thermoacoustic chillers that there is an optimal operating frequency in the thermoacoustic system, and the optimal operating frequency is related to pressure. In order to obtain the best cooling performance, pressure, operating frequency and cooling power should be combined; Islam^25^ pointed out that increasing the driving amplitude can effectively increase the cooling capacity. These results are consistent with the effect of optimal operating frequency and amplitude on the heat transfer of gas microclusters in thermoacoustic refrigerator proposed in this paper. In the initial stage of the design of thermoacoustic refrigerator, it is necessary to carry out fine theoretical calculation to determine the correlation between the target parameters such as cooling temperature and cooling capacity and the working parameters. While meeting the design objectives, the optimization of each parameter provides a theoretical basis for the manufacture of thermoacoustic refrigerator, which is also one of the significance of the model established in this paper in the field of thermoacoustic refrigeration.

**Table 1:** Parameters of gas column in resonant cavity.

Parameters	Permissible limits
${T}_{m} ({\text{K}})$	298.5
$\Pi$ (mm)	100
$\gamma$	1.4
$\rho (\mathrm{kg }\cdot{{\text{m}}}^{-3})$	1.25
${p}_{m}$(${\text{Pa}}$)	1 × 10^5^
$c (\mathrm{m }\cdot{{\text{s}}}^{-1})$	346
$h ({\mathrm{w }\cdot\left({{\text{m}}}^{2}\cdot\mathrm{ K}\right)}^{-1})$	80

## Influence of air mass amplitude, frequency and plate temperature gradient at different positions on heat transfer

In order to study the influence of various parameters on the heat transfer of gas, the resonator is normalized in the x direction, and *x* = *0* at the abdominal point of sound pressure. The length of the resonator is a quarter wavelength, and the width of the stack only affects the total amount of heat transferred, and does not affect the law of heat transferred under different parameters. In order to simplify the calculation, the width is set at 100 mm. The convective heat transfer coefficient is a process quantity, which is affected by many factors. Wang Qiuwang gave the range of convective heat transfer coefficient under several typical conditions in the book of Heat transfer^29^, where $h=80 {\text{w}}\,\cdot {({{\text{m}}}^{2}\cdot\mathrm{ K})}^{-1}$. The parameters in Table 1 are substituted into Eq. (12) for analysis.

**Figure 4:**
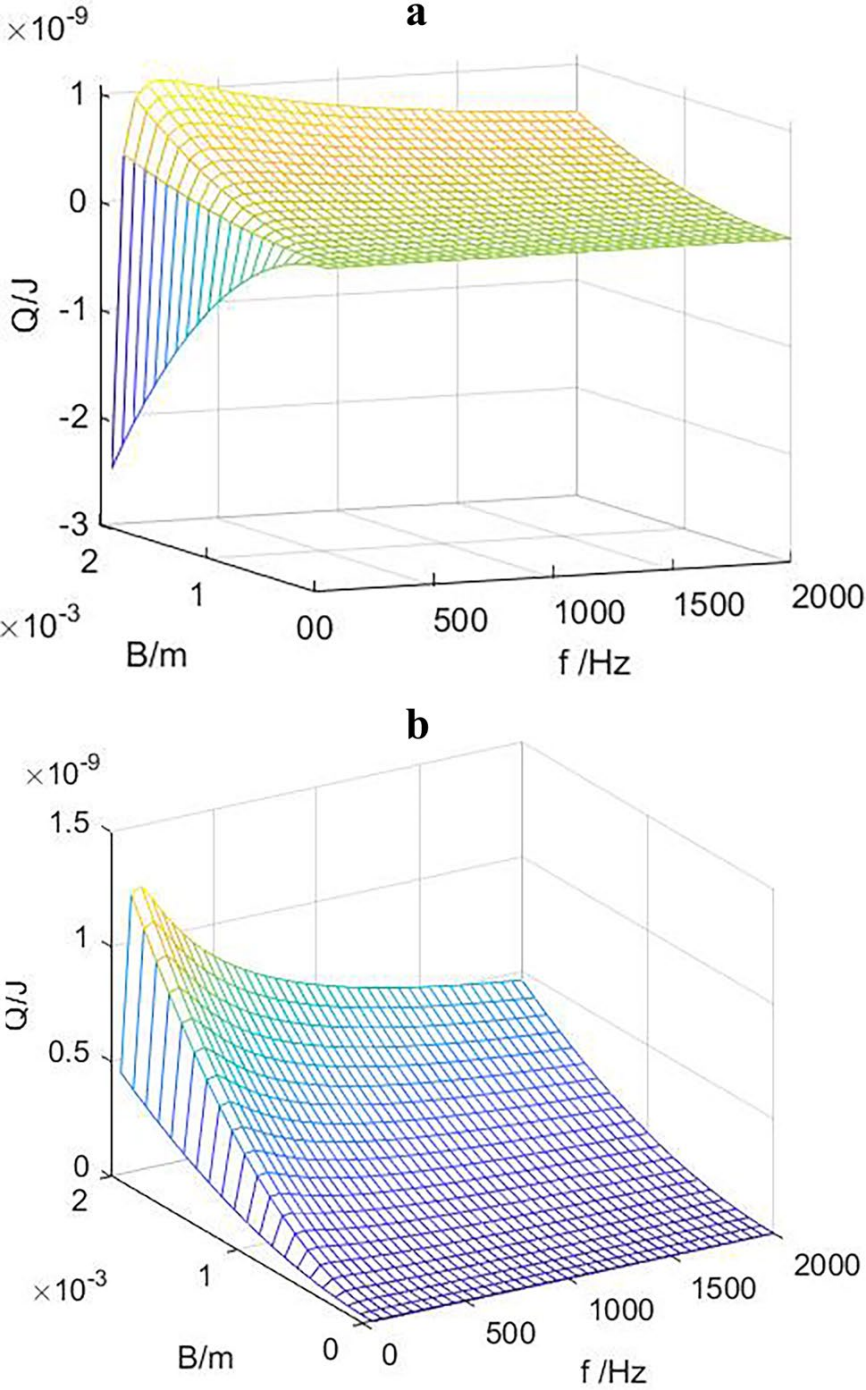
dTm/dx = 400 K/m, the variation of heat transfer with amplitude and working frequency at different positions of the stack (**a**) x_1_ = 0.5，(**b**) x_1_ = 0.3.

Figure 4 shows the relationship between amplitude, frequency and the heat transferred by gas microclusters at different positions of the stack. The heat transferred by increasing amplitude increases significantly with each working cycle. When x_1_ = 0.5 is located in the middle of the maximum and minimum pressure values, the system is in the stable working stage, there is a critical frequency f_crit_. when the working frequency is greater than fcrit, the moving heat is positive, the system can maintain a stable cooling state, but as the frequency increases, the heat moved will first increase and then decrease, the system has an optimal working frequency. If the operating frequency is less than fcrit, the system cannot cool under the required temperature gradient of the stack, and the heat of the stack is gradually transferred from the high temperature end to the low temperature end. When the system reaches equilibrium again, the temperature gradient of the stack decreases, and the cooling temperature decreases. For the same operating parameter, when x_1_ = 0.3, near the pressure belly point, the critical frequency disappears, and the optimal operating frequency still exists. With the increase of amplitude, the optimal frequency basically remains unchanged at a certain value. This is mainly because near the pressure belly point, the amplitude of the air mass is small, the pressure changes sharply, and the temperature changes greatly. In a amplitude range, the effect of the sound field on the heat transfer is greater than the influence of the temperature gradient of the plate, and the air mass can still move the heat at a lower frequency.

From the above analysis, increasing the vibration amplitude of the air mass can effectively improve the heat transfer capacity, so it is necessary to explore ways to increase the vibration amplitude of the gas to achieve large alternating flow of the gas column. The existing research results show that the sound intensity of piston modal vibration can reach tens of times that of standing wave sound field under the same excitation, and can produce alternating flow more intense than standing wave vibration, and the form of pressure field and velocity field generated by modal vibration is exactly the same as that of standing wave sound field. As long as the plate stack is placed in the appropriate position of the sound field, the effect of heat transfer can be generated, the effect is more significant, but also can greatly reduce the requirements of the loudspeaker, and by adjusting the structure of the resonator and the physical parameters of the working medium, the optimal working frequency can be consistent with the piston mode frequency, and the efficiency of heat transfer can be further improved.

**Figure 5:**
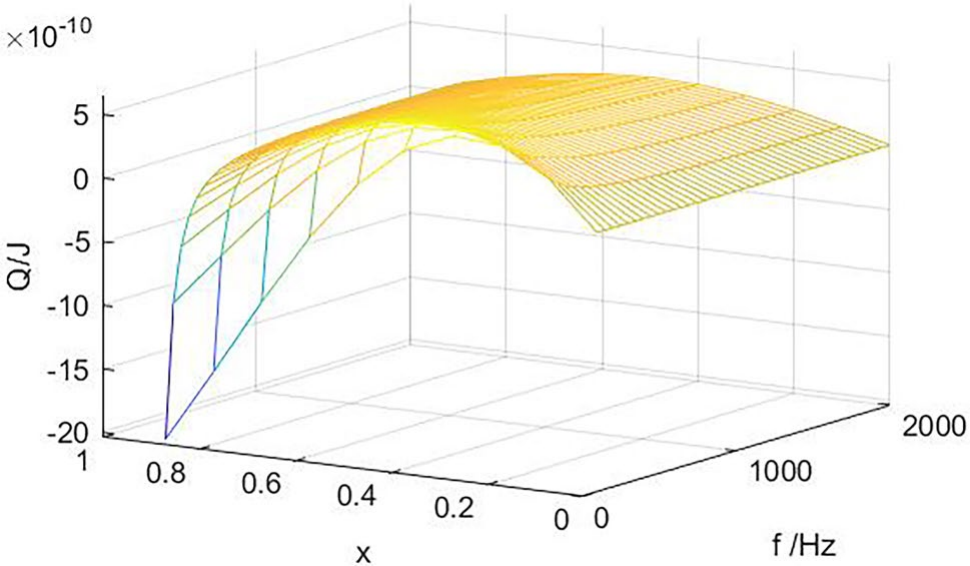
B = 1.5 mm, dTm/dx = 400 K/m, the relationship between heat transfer and working frequency at different positions.

Figure 5 shows the relationship between gas transfer heat and frequency at different positions of the sound field when the temperature gradient of the stack is constant. It can be seen from the figure that at x_1_ = 0.5, the heat transfer value of gas microclusters is the largest. When the stack temperature gradient is constant, it can be seen from Eq. (11) that the heat absorption of the air mass increases with the increase of P_1_ B, P_1_ B = 1/2 P^s^ B^s^ sin2kx, and the superscript s represents the absolute value of the sound pressure and amplitude, and the maximum value is obtained at x = λ/8 (λ is the wavelength), which is exactly x_1_ = 0.5 after the normalization of the resonator.

**Figure 6:**
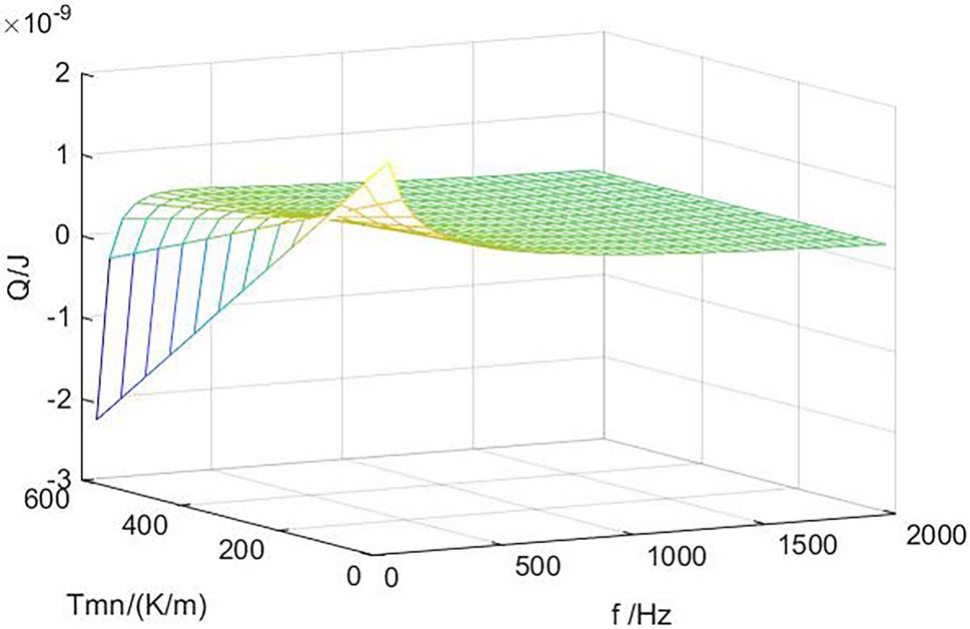
B = 1.5 mm, x = 0.5, the relationship between the heat transferred and the working frequency under different temperature gradients.

Figure 6 shows the relationship between heat transfer and working frequency under different stack temperature gradients. Different stack temperature gradients correspond to different critical operating frequencies. When *dT*_*m*_*/dx* is small, the heat transferred is positive, the system is in a cooling state, and there is no critical operating frequency, indicating that the effect of sound field on the air mass temperature is greater than that of the stack temperature gradient. With the increase of the frequency, the heat transferred under different temperature gradients first increases and then gradually decreases to a stable value, and the critical frequency also increases with the increase of temperature gradient. In practical applications, a large stack temperature difference is the goal to pursue, and the temperature difference between the two ends of the stack is affected by the stack temperature gradient and the stack length. When other conditions are certain, a smaller temperature gradient is conducive to the occurrence of thermoacoustic effect. The air mass moves more heat in one working cycle, and the cooling capacity of the thermoacoustic refrigerator is large. However, in order to obtain a large stack temperature difference and achieve a lower cooling temperature, it is necessary to increase the stack length or increase the stack temperature gradient. Therefore, the determination of the temperature gradient and length of the stack should be based on the working environment of the thermoacoustic refrigerator, and the requirements of the cooling capacity and cooling temperature should be considered comprehensively.

## Formation mechanism of large temperature difference between two ends of plate stack

As mentioned earlier, the temperature change caused by the sound pressure during the vibration of the air mass at a certain location is very small. The stack with an amplitude length is referred to as the short stack, and the temperature difference between the two ends can be expressed as:

14 $$\Delta T={T}_{g}-{T}_{d}$$

With the passage of working time, the temperature difference between the two ends of the short plate stack becomes larger and larger, and the gas temperature change T_1_ calculated by Eq. (2) becomes smaller and smaller, the effect of stack temperature gradient on gas temperature rise is close to that of sound pressure, until the system is in a stable working state, T_1_ no longer changes, Eq. (2) can be written as:

15 $$\upeta \frac{\gamma -1}{\gamma }\frac{{T}_{m}}{{P}_{m}} {p}_{1}=\frac{1}{j\omega }{u}_{1}\frac{d{T}_{m}}{dx}$$

$\eta \le 1$, proportional coefficient. Let ${T}_{s1}=\frac{\gamma -1}{\gamma }\frac{{T}_{m}}{{P}_{m}} {p}_{1}$, which is the temperature change of air mass under sound pressure, substitute ${\text{B}}=\frac{1}{j\omega }{u}_{1}$ into Eq. (14), and the temperature gradient of the short plate surface can be expressed as:

16 $$\frac{{{\text{dT}}}_{{\text{m}}}}{{\text{dx}}}=\upeta \frac{{{\text{T}}}_{{\text{s}}1}}{{\text{B}}}$$

When η = 1, the temperature gradient of the stack reaches the maximum, and Eq. (16) can be simplified as:

17 $$\frac{{{\text{dT}}}_{{\text{m}}}}{{\text{dx}}}=\frac{{{\text{T}}}_{{\text{s}}1}}{{\text{B}}}$$

Since the air mass at the equilibrium position has the same temperature distribution as the surface of the short stack, the gas above the entire stack actually has a temperature gradient similar to that of the stack. When working steadily, under the action of the sound field, each air mass synchronously moves the heat from one side of its equilibrium position to the other side to achieve pumping heat. Along the direction of sound wave propagation, the whole stack can be regarded as a series of multiple short stack, each of which has a length equal to an amplitude. According to the continuity of the stack temperature change, although the stack temperature change is small within an amplitude range, the temperature difference between the two ends of the stack also increases with the increase of the number of short plates in series, thus obtaining a significant temperature difference between the two ends of the stack. Obviously, the temperature difference between the two ends of the stack can be increased by increasing the stack length.

The air mass moves the heat from the low temperature end to the high temperature end, and the effect of the temperature gradient of the plate is just the opposite, because of the heat conduction inside the plate, a part of the heat is returned from the high temperature end to the low temperature end, so η < 1. The less heat leakage in a working cycle, the closer the η value is to 1, and the smaller the deviation between the stack temperature gradient and the result calculated by Eq. (16). Even if η = 1, the temperature difference between the two ends of the short stack is very small when the stack temperature gradient reaches the maximum.

## Conclusion

In this paper, a mathematical model of heat transfer by air mass in one working cycle is established, and the influencing factors in the model are analyzed. The idea of a short plate stack with a length of one amplitude is proposed, and the formation mechanism of the large temperature difference between the two ends of the plate stack is explained. The heat transfer process of single short plate stack and air mass is studied, and the following conclusions are obtained:

1. Under a certain working environment, the heat flux in the resonator can be obtained through the mathematical model of heat transfer by gas microclusters during one working period. In actual work, the heat flux is large, indicating that there is more heat flowing from the low temperature end to the high temperature end between the plates per unit time, which is conducive to the improvement of the cooling capacity. Therefore, the heat transfer in the mathematical model can be taken as the target of optimization design, and the variation law of heat flux between plates under different influencing factors is obtained, which provides a basis for the selection of parameters of thermoacoustic refrigerator.
2. Increasing the operating frequency does not necessarily increase the cooling capacity. There is an optimal operating frequency in the system. In order to obtain a higher cooling capacity, the system should work at the optimal frequency. When the system is in a stable working state, the thermoacoustic refrigerator has a critical operating frequency, and when the excitation frequency is greater than the critical operating frequency, the system can achieve refrigeration. With the increase of frequency, the heat transferred by the air mass first increases and then gradually decreases, and tends to a stable value. When the excitation frequency is equal to the critical frequency, there is a thermoacoustic effect, but the moving heat is zero, indicating that the influence of the sound field on the air mass temperature is the same as that of the stack temperature gradient, and there is no heat transfer between the air mass and the plate. When the excitation frequency is less than the critical frequency, the influence of the sound field on the air mass temperature is less than that of the stack temperature gradient, and the system cannot be cooled, and the cooling function needs to be restored after automatic adjustment to reach the equilibrium state after a period of time. At this time, the temperature gradient of the stack decreases, and the cooling temperature decreases accordingly.
3. The critical operating frequency is mainly affected by the stack temperature gradient (the temperature difference between the two ends of the stack) and the position of the stack in the sound field, and the critical operating frequency increases with the increase of the stack temperature gradient. With the pressure belly point as the reference point, the greater the distance from the reference point, the greater the critical operating frequency.
4. The amount of heat transferred is proportional to the square of the gas amplitude, which indicates the direction of efforts to improve the cooling capacity. In this paper, a new idea is proposed to increase the amplitude by replacing the standing wave vibration with the gas piston mode vibration. The excitation frequency is matched with the optimal heat transfer frequency and the first-order piston mode frequency of the gas column to achieve more efficient heat transfer, which provides a theoretical reference for the design and improvement of thermoacoustic refrigerator.
5. The temperature gradient of the plate and the position of the plate in the sound field have influence on the heat transfer. On the premise that the structure and physical property parameters of the stack are determined, the stack temperature gradient is mainly determined by the sound field and the working medium. If the stack temperature gradient is small, the stack length can be increased to increase the stack temperature difference. According to the mathematical model of heat transfer, the heat transfer is the largest at λ/8, but according to the linear thermoacoustic theory^9,27^, the sound power is proportional to the square of the sound pressure, the closer the pressure belly value point is, the greater the sound power is, and the maximum sound power is at the maximum pressure point. However, the air mass is not moving at the point of maximum pressure, only periodically changes temperature at its own equilibrium position, and can not achieve heat transfer, so the position of the plate in the sound field should be between 0 and λ/8, and the specific position should be determined to take into account the transfer of heat and sound power.
6. The idea of short plate stacking in series vividly explains the formation mechanism of the temperature difference between the two ends of the plate, establishes the relationship between the temperature difference between the two ends of the plate and the temperature gradient of the plate stacking and the amplitude of the sound field, and provides a design idea for improving the refrigeration efficiency of the thermoacoustic refrigerator and realizing low temperature refrigeration under certain conditions.

## Data Availability

All data generated or analysed during this study are included in this published article.
